# The role of athlete commissions in addressing sport-related human rights abuses: a case study of the Commonwealth Games Federation Athletes Advisory Commission

**DOI:** 10.1007/s40318-021-00208-9

**Published:** 2022-01-17

**Authors:** Urvasi Naidoo, David Grevemberg

**Affiliations:** Nottingham Trent University, Nottingham, UK

**Keywords:** Athletes’ Commission, Commonwealth Sport, Constitutional reform, Governance, Human rights

## Abstract

Athlete commissions have an important role to play in addressing sport-related human rights abuses. This article will explore this by examining the Commonwealth Games Federation (CGF) which made constitutional changes and created its first athlete representation body in 2017—the CGF Athletes Advisory Commission (AAC). These governance and legal changes empowered athletes to be part of the CGF leadership. Under the new governance structure, the Chair of the AAC is a voting member of the CGF Executive Board. The AAC met for the first time in 2018. In 2020, they launched their strategy (CGF Media Release 13 May 2020. https://thecgf.com/news/cgf-athletes-advisory-commission-launch-new-strategy. Accessed 28 October 2021) which seeks to uphold and live the Commonwealth Sport values of humanity, equality and destiny. The strategy identifies the Commonwealth athletes as agents of change, advocates for integrity and ambassadors for respect, impartiality and non-discrimination. How realistic is this ambition? The CGF itself, Commonwealth Sports Ministers and Commonwealth Member Countries have all adopted zero tolerance policies towards human rights violations (See The Commonwealth Consensus Statement on Promoting Human Rights in and through Sport 27 October 2020. https://thecommonwealth.org/sites/default/files/inline/CW%20Consensus%20Statement-ADOPTED%20v2.pdf. Accessed 28 October 2021 and The CGF Transformation 2022 Refresh 3 September 2019 Page 31. https://thecgf.com/sites/default/files/2019-10/CGF_TRANSFORMATION%2022_BROCHURE_FINAL_16-08-19_LOW%20RES.pdf. Accessed 28 October 2021). The authors will speak to the current Chair of the AAC and the previous Chair of the AAC to ask some pertinent questions around what impact the 2020 AAC strategy can have in safeguarding participants from sport related human rights abuses. The interview will explore policy change, governance reforms and long terms strategies which can maximize the role of athletes in protecting, respecting and fulfilling the human rights of not just athletes but all involved in the delivery of the Commonwealth Sport movement.

## Introduction

Since the turn of the century, and especially in the last 10 years, we have seen a huge growth in the use of the athlete voice as it becomes crucial for athletes to stand up for human rights. Whether it be fighting for better safeguarding, promoting athlete welfare, speaking up against wrong-doing or holding true to the values of equality and inclusion, athletes have realised what powerful activators they can be and how they can truly make a difference.

The first meeting of the IOC Athletes’ Commission was in 1982.^1^ The formation was part of a strategy to modernise the Olympics, but it is documented that the IOC President appointed the members for the early years, and the impact of the Commission was limited. Pursuant to the recommendation of the IOC 2000 Reform Commission that “athletes should be well represented at all levels of the sports movement: IOC, IFs, NOCs and NFs”,^2^ the IOC Athletes’ Commission was re-constituted. At this point, the members became IOC Members and gained representation on the IOC Executive Board. However, their impact is still in question as recently discussed in relation to the controversial enforcement of Rule 50.2 of the Olympic Charter and its subsequent amendment on eve of the Tokyo Olympics.^3^^,^^4^^,^^5^

The Commonwealth Games Federation (CGF) only created its’ athlete body, the CGF Athletes Advisory Commission (AAC) in 2017. In this essay, we use the case study of the CGF to examine the role of athlete commissions in addressing sport-related human rights abuses.

The authors will speak to the current Chair of the AAC and the previous Chair of the AAC to ask some pertinent questions exploring what impact the 2020 AAC strategy can have in safeguarding participants from sport related human rights abuses.

## CGF Human Rights Commitment

The Commonwealth Games and Commonwealth Sport Movement have a well-established history and proud heritage of uniting diverse nations and cultures through the power of sport.

Glasgow 2014 embraced a broad Commonwealth conversation on respecting, protecting and promoting human rights, aligning its planning and delivery in close adherence with the United Nations Guiding Principles on Business and Human Rights. The Organizing Committee published its approach and results in pre- and post-games reports;^6^ the first mega-sporting event to do so anywhere in the world. This was largely applauded by sport and human rights bodies worldwide and continued to be used as a benchmark in CGF work for both Gold Coast 2018^7^ and Birmingham 2022.

In 2017, a Human Rights Statement was adopted by the CGF Executive Board to cement the CGFs commitment to embed human rights within CGF governance, management systems, development, events, fundraising and marketing.^8^

In 2018 at the CGF General Assembly, the CGF published a guide for mega sports events “Championing Human Rights—In Governance of Sports Bodies”. The guide was developed by a taskforce, which was chaired by David Grevemberg, CGF Chief Executive Officer (2014–2021), and David Rutherford, New Zealand's Human Rights Commission Chief Commissioner (2011–2018).^9^

Sports Bodies have a responsibility to respect human rights: that is, to provide equal opportunity to play and to avoid people’s human rights being harmed through their activities or business relationships and to address harms that do occur. At all times, sports bodies should strive to act responsibly, through their governance, through proper safeguarding, and through respecting the rights of all stakeholders including athletes, fans, communities, workers, children, volunteers, journalists, human rights defenders and potentially marginalized groups.^10^

The Gold Coast Commonwealth Games 2018 celebrated the largest fully inclusive Para-Sport programme ever and was the first games to achieve gender equality in terms of medal events. It also championed LGBTI+ inclusion. It was the first major sporting event in Australia to include a Reconciliation Action Plan.^11^

In 2019 at the CGF General Assembly, a workshop was held for delegates to outline ways in which integrating human rights could produce stronger frameworks for good governance and exchange case study examples and ideas to inspire the CGAs across the Commonwealth to integrate human rights and advocate for change. Discussions stemmed around implementing a projects, respect and remedy framework and working together with the CGF to inform a long-term human rights strategy.

In October 2020, Commonwealth member countries unanimously adopted a statement to promote human rights and tackle discrimination at all levels of sport from community games to elite sporting events. The consensus statement sets out 13 commitments and serves as a guide to protect and promote the human rights of every individual involved in the sport sector.^12^

Each of the Commonwealth’s 54 member countries has committed to combating racism within the sport sector, promoting women’s equality and empowering people with disabilities to participate on an equal basis.

Through the statement, member countries have agreed to implement a zero-tolerance policy towards violence, harassment, abuse and discrimination in sport, while setting up multi-sectoral platforms to respond to human rights violations.

One emphasis is on introducing human rights objectives in the life cycle of major sporting events to spur a lasting positive legacy from bidding, planning and procurement to competition delivery. The CGF has responded by ensuring that the organising committee of Birmingham 2022 Commonwealth Games has a human rights strategy in place.

In 2020, during the height of advocacy and activism of the “Black Lives Matter” Movement and the global reckoning on racial equality, the CGF took a stand. CGF President Dame Louise Martin and CEO David Grevemberg published an open letter acknowledging that citizens across the world had mobilized to stand up for equal rights, for freedom, fairness, equality and justice. CGF called for action stating that sport must use its voice and continually seek to reduce inequalities and build peaceful communities. This was also a wake-up call and granted CGF a social and moral license to start making changes to address these issues and others in the Commonwealth Sport Movement.^13^

These examples showcase how the Commonwealth Sport Movement has responded to the realities and challenges of the past and present to contribute to a freer, fairer, more equal and just tomorrow for the people and communities that it represents and serves.

## New Athletes Commission

In 2016, constitutional changes were made to create an athlete representation body, the AAC. These governance and legal changes empowered athletes to be part of the CGF leadership. Under the new governance structure, the Chair of the AAC became a voting member of the CGF Executive Board. The AAC structure also ensures that there is athlete representation from all six Commonwealth Regions and that there is gender parity. This step greatly increased the Movement’s alignment with the voices of Commonwealth Athletes. The strategic focus of the AAC in the lead up to Birmingham 2022 and beyond will be critical in ensuring athlete health, safety and wellbeing remains a top priority, particularly recognising the global challenges of enabling sport to fully return after the COVID-19 pandemic. Additionally, many critical issues facing athlete rights and representation, athlete advocacy and activism and athlete licensing and content will continue to be at the forefront of future dialogue and Movement-wide initiatives.

## New strategy

In May 2021, the ACC launched its own Strategy^14^ outlining a commitment to ensure Commonwealth athletes are inspiring leaders, agents of change, advocates for integrity and ambassadors for respect, impartiality and non-discrimination. It also indicates how athletes will play a leading role in shaping all Commonwealth Sports Movement decisions. Fully aligned to the CGF values of humanity, equality and destiny and the CGF strategy Transformation 2022.

The Commonwealth athletes stand for much more than a number on a bib, or a name on a start list. They have an important role to reach further than their medal or their personal best. They are the vanguard of a great movement whose purpose has been carried and shared across decades, across generations and across borders.^15^

## Interview

The authors interviewed the current Chair of the CGF Athletes Advisory Commission Brendan Williams (BW) and the previous and first Chair, Rhona Toft (RT).

Former high jump star Brendan Williams of Dominica was confirmed as the new Chair of the CGF Athletes Advisory Commission (AAC) in March 2021, becoming the first Caribbean athlete in history to lead the athletes’ commission of a major International Sports Federation. He competed for Dominica in the Commonwealth Games at Delhi 2010 and Glasgow 2014 and was the Caribbean representative on the CGF AAC since its formation in 2018. Brendan is also Chair of the Dominica Athletes’ Commission, a Board member of the Dominica Olympic Committee and serves on the athletes’ commission of the Caribbean Association of National Olympic Committees (CANOC).

Rhona Toft is Scotland’s most decorated and leading hockey goal-scorer of all time, having competed for her country in hockey at the 1998, 2002 and 2006 Commonwealth games and for Great Britain at the Atlanta 1996 and Sydney 2000 Olympic Games. Rhona is also an experienced hockey coach, taking the Great Britain Youth Olympic Squad to a Gold medal at the Youth Olympics in 2009. She served as Chair of the AAC from April 2018 to March 2021. She is currently Chair of World Netball “Voice of the Athlete” Working Group.

The interview was semi-structured in that open ended questions were explored with the interviewees. Informed consent was obtained.

### CGF commitment is to use the power of sport to respect, protect and promote human rights, uphold the highest standards of integrity and transparency and nurture long-term sustainable development in all that it does—How important is the work that the AAC is doing?

BW*:* Athletes can make a difference. In Tokyo, we saw much more prominence around the athlete voice. Athletes taking a stand against racial disparity and standing up for LGBTQi. Most notably with Raven Saunders raising the cross over her head to signify her support for human rights.^16^ I see the AAC as empowering the athletes by drafting and implementing the CGF Athletes Advocacy Policy. It is important that the athletes can stand up whilst also respecting the Commonwealth Values and their own values.

RT: The Athlete voice has moved forward significantly since 2014 when the AAC was at its beginning. I agree that voice is necessary but doing it in a manner which is respectful and not confrontational and in keeping with the values is also important. This will become more prominent, and the AAC strength is in having a coordinated approach, and everyone standing behind the agreed position.

### The Chair of the AAC has a seat on the Executive Board but the CGF has also now started to appoint an athlete to all CGF commissions and committees what does this mean?

BW: For me, it is a move away from “athlete focused” to “athlete centered”. Athlete focused being decisions made for the benefit of the athletes, whereas centered meaning athletes involved in the actual decision making because they are the main beneficiaries of the policies and decisions. Athletes are now front and center.

RT: In the old days, I think there may have been hesitance to involve athletes, athletes were just supposed to turn up and perform but that has changed, and athletes are now engaged and involved. It’s also a big change for the athletes, when you are competing you are not so interested in governance but if there is better education and involvement then athletes can have a better impact on their organisations, sport is just the beginning—athletes go on to be part of other processes.

BW: It is a truly holistic approach. The athletes are the biggest stakeholder, so their involvement leads to better decision making.

### What do you perceive as the biggest challenges for the AAC in the lead up to Birmingham 2022 Commonwealth Games?

BW: The implementation of policies especially the Athlete Advocacy Policy, the policies need to be implemented properly. Also, to get acceptance of the athlete voice across all the CGA’s. There are some where they are there but are not pivotal in decision making.

RT: In relation to lead up to Birmingham—mental health and athlete well-being has become very prominent. All athletes face pressure and anxiety but now they feel able to speak up about it. It is going to come up more and how the CGF responds and supports athletes will be key, it is difficult to define as it is not just pressure.

BW: That is true all athletes put themselves under pressure to perform. Another pressure I discovered is the monetary benefit with some countries paying their athletes money for winning a medal.

### What about issues in relation to inclusion and diversity?

BW: We need to be welcoming and respect all athletes. Clearly in Tokyo, there were issues regarding the transgender athletes and the athletes subject to testosterone restrictions. We will be informed by the medical science when it relates to performance, we need to be ensuring fairness as well as respect for everyone. It cannot be based on feelings and emotions.

RT: I think a lot more research is needed to inform the medical science. It must be fair competition and that may come down to individual sports.

### The AAC are tasked with empowering and equipping athletes as agents of change and advocates for integrity—What more can the CGF do?

BW: We have limited resources and meet once a month, and it is not enough to fulfil the mandate of the AAC. If we had an officer, they could complete work in between meetings and go about implementing the policies. The voluntary nature of the structures makes progress slower.

RT: It is a big job, and the demand and work are growing especially as AAC members now sit on other commissions and committees as well. That would be another pointer toward being truly athlete centered—if the CGF were to further resource the AAC it would be a strong sign that the CGF is backing the athletes.

BW: Having the mechanism to bring things to fruition and maintain the momentum.

RT: Transformation 2022, sought to ensure all CGAs have a meaningful athletes representation structure with an athlete rep on every CGA Board, and we are very close to achieving that, if the CGF could do more to attain this it would be a very good milestone.

BW: I would love to be able to call on 72 athlete reps across all CGAs.

### What do you see in relation to beyond Birmingham?

BW: We will need to develop a new AAC strategy. For various reasons, not all strands of the 2022 strategy have been achieved, and we need to redefine the priorities and put in place something which is fully athlete centered and embraced by the CGF.

RT: it is important to see commitments through to completion as that builds trust and confidence, but it also comes back to the resource issue. There are a lot of positives that can be drawn on, and we do know that things take time, as we have said, due to the voluntary nature of the structures, so we need to have realistic timeframes. 2022 was an ambitious strategy, and we did not anticipate the pandemic and all other factors.

BW: Yes, I just conclude by saying everything needs to be holistic in terms of not making decisions for the athletes but rather empowering athletes to make decisions. This needs to be consistent, so all year round and in between games as well. Building on the existing good work of the CGF.

RT: Yes, I would just say that the CGF has a fantastic group of athletes on the AAC and needs to maximise that opportunity and maintain the momentum using the strengths, experience and energy of the AAC members. Constant re-evaluation to ensure we are leading in terms of best practice.

## Conclusion

This study has provided some insight into the CGF and the AAC. Several key findings have emerged.

Firstly, the CGF has expressed strong commitment to human rights throughout all its activities. Throughout the Commonwealth Sport movement, sports bodies are encouraged not just to respect human rights and prevent causing harm but also to embed this within governance and operations.

Secondly, the CGF is committed to developing an athlete centric way of operating, empowering athletes by giving them a voice on the Executive Board and other decision-making Committees and Commissions. Confines of space have not allowed an in-depth discussion into all the human rights issues facing the AAC, but we have established that the CGF has a strategic commitment to representation, inclusion, freedom of expression and to listening to the athlete voice.

Finally, whilst the CGF has taken important steps since the first AAC meeting at the Gold Coast in 2018, the momentum must now be maintained to achieve true athlete representation and the power to influence decision making. The voluntary nature of the structures puts limitations on the AAC and to progress at a pace the AAC needs to be properly resourced and supported by the CGF.

It would be interesting to undertake further analysis after Birmingham 2022 to gain insight into the impact the AAC had on policy and decision-making processes for the event. It is anticipated that the next AAC strategy 2022–2026 will continue to pursue an athlete-centred, sports-focused movement that is fully aligned to the values of humanity, equality and destiny.

## Footnotes

1 See IOC Website 11 April 2019. https://olympics.com/ioc/news/a-growing-voice-the-history-of-the-ioc-athletes-commission. Accessed 28 October 2021.

2 See Report by the IOC 2000 Commission to the 110th IOC Session 12 December 1999 Recommendation18 Page 13. https://stillmed.olympics.com/media/Document%20Library/OlympicOrg/IOC/Who-We-Are/Commissions/IOC-2000-Reform-Follow-up/EN-110th-IOC-Session-Report-by-the-IOC-2000-Commission-December-1999.pdf. Accessed 28 October 2021.

3 See Inside the Games Global Athlete call on IOC and IPC to immediately abolish Rule 50 14 June 2020. https://www.insidethegames.biz/articles/1095305/global-athlete-rule-abolition. Accessed 28 October 2021.

4 Elsborg, S Page 44–48.

5 See IOC Rule 50.2 Guidelines for Tokyo 2020. https://olympics.com/athlete365/app/uploads/2021/07/Rule-50.2-Guidelines-Olympic-Games-Tokyo-2020-Final.pdf. Accessed 28 October 2021.

6 See Glasgow 2014 Approach to Human Rights booklet 2014. http://www.glasgow2014.com/sites/default/files/documents/Glasgow%202014%20-%20approach%20to%20human%20rights%20-%20December%202013.pdf. Accessed 28 October 2021 and Scottish Human Rights Commission news article 2 June 2014. https://www.scottishhumanrights.com/news/scotland-leads-commonwealth-in-developing-human-rights-policy-for-games/. Accessed 28 October 2021.

7 See Gold Coast Approach to Human Rights Booklet 2018. https://gc2018.com/sites/default/files/2018-08/GC2018%20Approach%20to%20Human%20Rights%20and%20Post-Games%20report%20-%20August%202018.pdf. Accessed 28 October 2021.

8 See CGF website Human Rights Policy Statement 5 October 2017. https://thecgf.com/sites/default/files/2018-03/CGF-Human-Rights-Policy-Statement-17-10-05.pdf. Accessed 28 October 2021.

9 See Inside the Games article 2 April 2018. https://www.insidethegames.biz/articles/1063350/cgf-publish-mega-sports-guide-on-boosting-human-rights-in-sport. Accessed 28 October 2021

10 Championing Human Rights in the Governance of Sports Bodies March 2018. https://www.insidethegames.biz/media/file/102424/Championing_Human_Rights_in_the_Governance_of_Sports_Bodies,_MSE_Platform.pdf. Accessed 28 October 2021.

11 Gold Coast 2018 Reconciliation Action Plan 2018. https://gc2018.com/rap. Accessed 28 October 2021.

12 See 2 above The Commonwealth Consensus Statement on Promoting Human Rights in and through Sport 27 October 2020.

13 See CGF media release An open letter to sport #BlackLivesMatter 2 June 2020. https://thecgf.com/news/open-letter-sport-blacklivesmatter. Accessed 28 October 2021.

14 See 1 Above CGF Media Release 13 May 2020.

15 See 2 Above The CGF Transformation 2022 Refresh 3 September 2019 page 35.

16 See BBC news 2 August 2021. https://www.bbc.co.uk/news/world-us-canada-58048727. Accessed 28 October 2021.
